# Botulinum toxin in the management of acquired motor fusion deficiency: The missing links

**DOI:** 10.4103/0301-4738.67046

**Published:** 2010

**Authors:** Pramod Kumar Pandey, Subhash Dadeya, Pankaj Vats, Anupam Singh, Neha Rathi, Sonal Dangda

**Affiliations:** Guru Nanak Eye Center, Maulana Azad Medical College, University of Delhi, New Delhi-110 002, India

Dear Editor,

Murthy *et al*.[1] conflate central motor fusion deficiency [CFD] in a young woman who had suffered a head injury in the past and report excellent recovery following botulinum toxin injections to both lateral recti. As the entity reported upon is rare and often a diagnosis of exclusion, we feel certain comments are in order. Entities like internuclear ophthalmoparesis [INO], convergence/ accommodative paresis, saccadic intrusions/oscillations as well as a preexisting decompensated phoria or a monofixation syndrome could create diagnostic confusion.[2,3] Unmistakable adduction deficit in both eyes on versions[1] a finding, never reported with CFD, invariably indicts bilateral INO. Lack of convergence and a large exodeviation further buttress that contention and/or an accommodative/ convergence palsy. A decrease in binocular visual acuity following head shaking, if found, might have been vital input for bilateral medial longitudinal fasciculi [MLF] involvement as is seen in bilateral INOs.

CFD, a rare entity, defies neuroanatomic correlation; it is often diagnosed anecdotally and subjectively on the basis of the inability to fuse two images in free space or on a haploscopic device, in the absence of any objective criteria. Invariably a small hypertropia, bilateral extorsion along with vertical bobbing of nonfusible image on attempted fusion are reported.[4] None are however documented here[1] and militate against such a diagnosis. Above fellow travelers consistently seen in CFDs, covertly implicate a pretectal and/or bilateral MLF involvement. As magnetic resonance/ diffusion tensor imaging was not done,[1] a bilateral MLF lesion might have been overlooked.[5] Foveal visual targeting with binocular fusion and stereo-acuity requires highly synchronous eye movements that place object of regard on corresponding points of both retinas. It is brought about by precise coordination between the 3^rd^, 4^th^ and 6^th^ cranial nerve nuclei and their inter-neuronal pathways projecting through the rostral interstitial nucleus of MLF, posterior commissure, both MLFs and para pontine reticular formation. MLF also receives vestibular inputs and modulates central vestibular tone. As both MLFs are close to each other, bilateral direct/indirect injury is common and may be overlooked as it has a limited yield on magnetic resonance imaging (MRI).[5] Similar central vestibular tone imbalance may also follow prolonged visual deprivation resulting from decreased afferent input from one of the optic nerves[6] and may lead to sensory deviations, dissociated deviations or CFDs. A pretectal or bilateral MLF etiology may also generate a CFD-like picture from a coexisting horizontal arm of the skew deviation or saccadic intrusions. CFD thus likely may veer around a pretectal or MLF localization with an imbalance in the central vestibular tone as primary pathology and may follow diverse etiologies, both central [head injury] and peripheral [visual deprivation].

Excellent results/recovery reported with botulinum toxin injection with restoration of fusional amplitudes[1] further betray an alternative diagnosis of bilateral INO, skew or convergence palsy rather CFD. Restoration of fusion in CFD is rarely achieved, so much so that surgery is not recommended for such cases.[2] Extended orthoptic exercises and prisms over long periods of time have a limited role in some cases with preexisting intermittent fusion.[2,4] As botulinum toxin works at the peripheral level it can not be expected to restore fusion in CFD miraculously over and above prisms and orthoptic exercises.
